# Optimizing Surgical Outcomes in Congenital Syndactyly: Evaluation of Flap and Graft Techniques in Older Pediatric Patients

**DOI:** 10.7759/cureus.64559

**Published:** 2024-07-15

**Authors:** Man D Phan, Quoc H Truong, Truc T Ho Nguyen, Duyen M Ngo Thi, Phi D Nguyen

**Affiliations:** 1 Orthopaedic Surgery, Pham Ngoc Thach University of Medicine, Ho Chi Minh City, VNM; 2 Pediatric Orthopaedics, Hospital for Traumatology and Orthopaedics, Ho Chi Minh City, VNM; 3 Anaesthesia, City Children’s Hospital, Ho Chi Minh City, VNM; 4 Orthopaedics, Burns, Plastic Surgery, City Children’s Hospital, Ho Chi Minh City, VNM

**Keywords:** web spacing, surgical outcome, grafting, flap techniques, congenital syndactyly

## Abstract

Background

Congenital syndactyly is a common congenital hand anomaly that impairs daily activities and impacts both functional and aesthetic outcomes. The fusion of adjacent fingers limits functionality and often requires surgical intervention to restore web spacing, maintain function, and improve appearance. This study evaluates surgical outcomes of congenital syndactyly treatment using flap and graft techniques, focusing on older patients.

Methodology

This study utilized retrospective data collected from patients aged 2 to 12 years diagnosed with congenital syndactyly. These patients underwent surgical separation procedures employing various flap techniques and full-thickness skin grafts. The chosen methods aimed to minimize scarring, secure optimal blood supply, and reduce postoperative complications. Postoperative assessments included web spacing, aesthetic appearance, and functional recovery.

Results

Patients generally experienced improved web spacing and proper alignment, with minimal contracture post-surgery. Flap and graft techniques effectively reduced visible scarring and provided favorable cosmetic results. Functional recovery was significant, allowing patients to resume age-appropriate tasks with minimal limitations, thereby restoring confidence in daily activities. Despite not undergoing early surgery, older patients still achieved marked improvements in web spacing, aesthetics, and overall function.

Conclusions

Surgical treatment of congenital syndactyly using flap and graft techniques significantly enhances both functional and aesthetic outcomes, even when the intervention is delayed beyond the recommended early age. Comprehensive planning and tailored approaches are crucial to achieving optimal web spacing, minimized scarring, and restored hand function. These measures ultimately improve the quality of life for patients, regardless of age at the time of surgery.

## Introduction

Congenital syndactyly is a common congenital malformation, occurring in about 1 in every 2,000 live births globally [1]. It is characterized by the fusion of two or more adjacent fingers, resulting in functional limitations and aesthetic challenges. This anomaly is primarily attributed to disruptions in the fetal developmental process during the sixth to eighth weeks [2]. The condition can range from simple webbing to complex bone and soft tissue fusion, affecting daily activities and psychosocial well-being [3].

Surgical separation of fused fingers is the standard treatment to restore normal anatomy and improve both functional and cosmetic outcomes [4]. Various surgical techniques, including flap reconstruction and grafting, have been developed to separate the fused digits and create natural-looking interdigital web spaces. The use of these techniques aims to achieve adequate spacing, prevent scar contractures, and optimize blood supply to the reconstructed areas [5].

Despite advancements in surgical techniques, achieving consistent and high-quality outcomes remains challenging due to the complexity of syndactyly variations and patient-specific factors such as age, severity of the condition, and associated comorbidities. The timing of surgery, choice of technique, and postoperative care are crucial to reducing complications and ensuring a positive prognosis [6]. This study aims to evaluate the outcomes of surgical treatment for congenital syndactyly in children over two years old, utilizing flap and graft techniques. The focus is on the effectiveness of these methods in achieving functional and aesthetic improvements.

## Materials and methods

This retrospective study was conducted at our institution from 2017 to 2022, involving a total of 52 patients with congenital syndactyly who underwent surgical separation using flap and graft techniques. This study received approval from the Ethics Committee of the Hospital for Traumatology and Orthopaedics (approval number: 07632). The patients’ ages ranged from 2 to 12 years, with an average age of 6.4 years. All patients were evaluated for the type and severity of their condition before surgery, ensuring that those with congenital syndactyly affecting one or more digits and who were available for a minimum follow-up period of 12 months postoperatively were included. Patients with syndactyly as part of a syndrome involving other congenital abnormalities or those with previous hand surgeries that affected their syndactyly condition were excluded.

**Figure 1:**
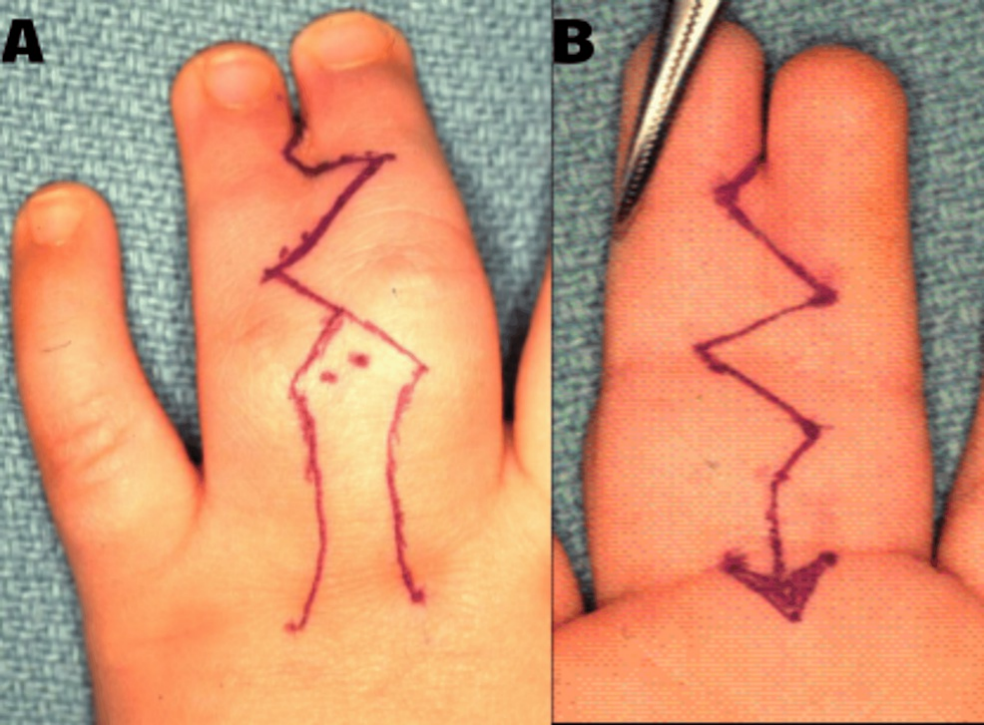
Flap design in one case. Original images from the study. A: Dorsal markings. B: Volar markings.

Preoperative assessment included a comprehensive clinical evaluation, including physical examination and radiographic imaging, to determine the extent and type of syndactyly. Patients were classified based on the type (simple or complex) and severity of syndactyly such as fusion of bones and soft tissue, with finger axis deviation. Various flap techniques were employed for reconstructing interdigital web spaces, with the choice of technique tailored to the patient’s anatomical needs to achieve optimal web spacing while minimizing scarring and contracture risks (Figure 1).

**Figure 2:**
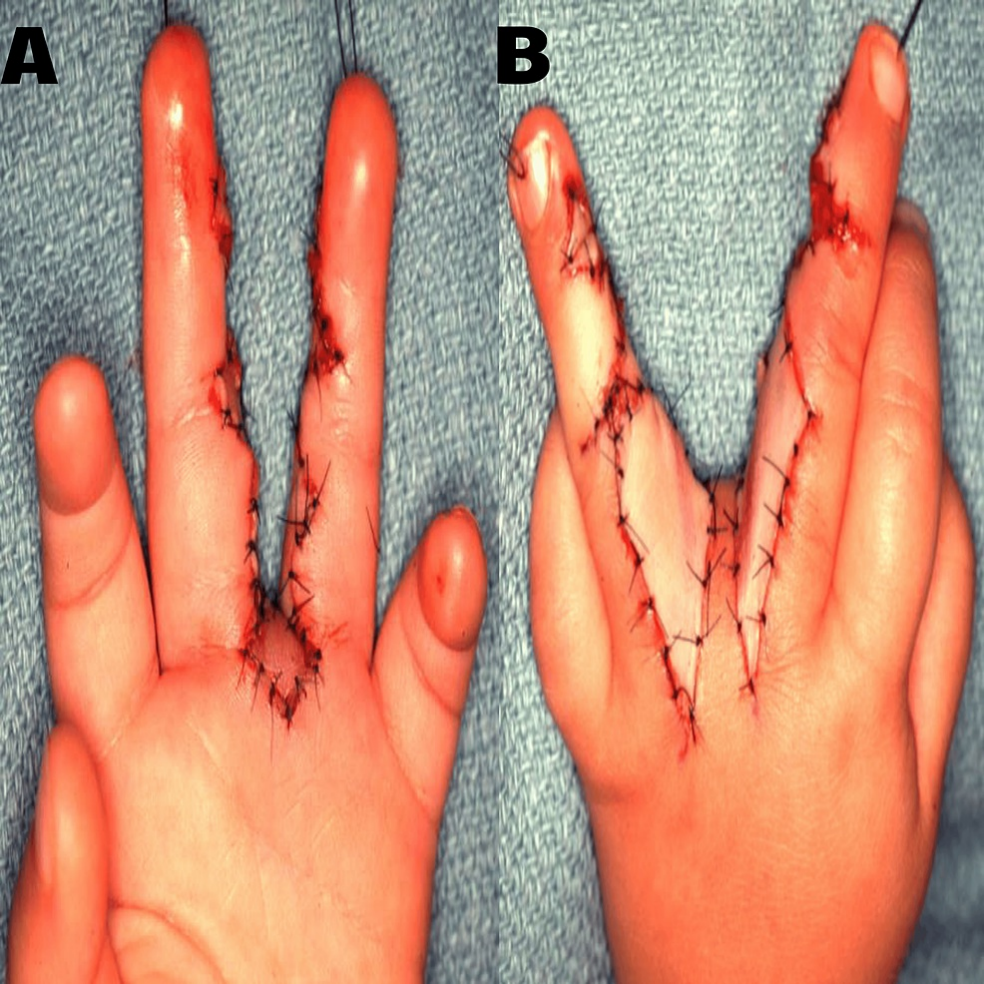
After skin graft in one case. Original images from the study. A: Volar view. B: Dorsal view.

When local tissue was insufficient to cover the reconstructed web spaces, full-thickness skin grafts from the patient’s thigh or groin were harvested. These grafts ensured complete coverage, adequate blood supply, and improved cosmetic results (Figure 2).

Postoperative care involved the immediate application of a protective splint following surgery to immobilize the fingers and mitigate the risk of accidental injury. Patients were provided with a customized rehabilitation plan aimed at promoting healing and restoring hand functionality. Regular follow-up appointments were scheduled to monitor the healing process, identify potential complications, and evaluate both functional and aesthetic outcomes. Outcome measures included the distance between reconstructed web spaces to assess the success of separation and prevent contractures, as well as cosmetic outcomes evaluated through our modified Global Aesthetic Improvement Scale (GAIS) (see Table 1), which considers the degree of scarring and the appearance of the reconstructed web spaces.

**Table 1:** Global Aesthetic Improvement Scale (GAIS) and our modification for syndactyly.

Rating	Description	GAIS modified for syndactyly
5 = Much improved = Excellent	Marked improvement in appearance from the initial condition, touch-up treatment(s) is not indicated	Natural appearance of the web space
4 = Improved = Good	Obvious improvement in appearance from the initial condition, but a touch-up or re-treatment is indicated	Mild scarring, acceptable appearance
3 = No change = Fair	The appearance is essentially the same as the original condition	Moderate scarring, noticeable appearance
2 = Worse = Poor	The appearance is worse than the original condition	Severe scarring, unsatisfactory appearance
1 = Much worse = Very poor	The appearance is much worse than the original condition	Necrosis, graft failure

Functional assessment was conducted using age-appropriate tasks to measure the mobility and dexterity of the separated digits, based on the Manual Ability Classification System (MACS) (see Table 2), with satisfactory results defined as achieving MACS level 1 or level 2.

**Table 2:** Manual Ability Classification System (MACS).

MACS level	Description
I	Handles objects easily and successfully
II	Handles most objects but with somewhat reduced quality or speed of achievement
III	Handles objects with difficulty; needs help to prepare or modify activities
IV	Handles a limited selection of easily managed objects in adapted situations
V	Does not handle objects and has severely limited ability to perform even simple actions

Statistical analysis was performed to evaluate the significance of the results. Percentages were used to present the proportion of patients achieving specific outcomes. All statistical parameters are included in the tables, enhancing the credibility and transparency of the data.

## Results

The study included 52 patients with congenital syndactyly who underwent surgical separation using flap and graft techniques. The patients’ ages ranged from 2 to 12 years, with an average age of 4.7 ± 2.3 years. The characteristics of the patients are detailed in Table 3.

**Table 3:** Patient demographics.

Characteristic	Number of patients (N = 52)	Percentage (%)
Age (mean ± SD)	4.7 ± 2.3 years	-
Gender		100
Male	29	55.8
Female	23	44.2
Type of syndactyly		100
Simple	34	65.4
Complex	18	34.6
Number of affected hands		100
Unilateral	31	59.6
Bilateral	21	40.4

Surgical outcomes

A total of 45 (94%) patients achieved satisfactory web spacing after surgical separation, with only three patients requiring secondary procedures due to minor contractures (web creep). Aesthetic outcomes are presented in Table 4. The majority of patients achieved either good or excellent aesthetic outcomes.

**Table 4:** Aesthetic outcomes post-surgery based on the modified Global Aesthetic Improvement Scale.

Score	Description	n (%)
5 (Excellent)	Natural appearance of the web space	30 (57.7%)
4 (Good)	Mild scarring, acceptable appearance	15 (28.8%)
3 (Fair)	Moderate scarring, noticeable appearance	5 (9.6%)
2 (Poor)	Severe scarring, unsatisfactory appearance	2 (3.8%)
1 (Very poor)	Necrosis, graft failure	0 (0.0%)

Functional assessment revealed that 90% of patients regained full dexterity and mobility, with the ability to perform age-appropriate tasks. The remaining patients had mild limitations but showed significant improvement compared to their preoperative status. Overall, the results indicate that surgical separation of fused fingers using flap and graft techniques effectively improves web spacing, aesthetic appearance, and functionality in children with congenital syndactyly.

## Discussion

Congenital syndactyly poses a significant challenge for hand surgeons due to its wide variability in presentation and the intricate anatomy involved. This study aimed to evaluate the effectiveness of flap and graft techniques in restoring function and aesthetics. The results indicate that these surgical techniques, tailored to the specific needs of each patient, yield satisfactory outcomes in most cases. In this discussion, we explore these findings and their implications, comparing them with existing literature, while identifying areas for further improvement. Proper interdigital web spacing is a critical indicator of surgical success, as it directly impacts hand function and appearance. In this study, 45 (94%) patients achieved satisfactory web spacing, consistent with findings from previous research that highlight the effectiveness of flap techniques for syndactyly correction [7]. However, three patients required secondary procedures to address contracture, demonstrating that while flap methods minimize scarring, meticulous postoperative care remains essential [8].

Z-plasty remains a popular technique due to its versatility and ability to redistribute tension across the web space. Nonetheless, other methods, such as rotational or triangular flaps, have also proven effective [9]. The choice of technique should be guided by the patient’s specific anatomy and the nature of the syndactyly [10]. A combination of different flap designs can further enhance web reconstruction, ensuring adequate coverage and reducing the likelihood of contractures [11]. Aesthetics are a major concern for patients and families, as the appearance of the hand significantly impacts self-esteem and social interactions. The majority of patients in this study achieved good or excellent aesthetic outcomes, consistent with other reports highlighting the benefits of individualized flap techniques [12]. The minimal visible scarring and natural contouring of the web spaces contribute to high patient satisfaction. However, cases with moderate scarring still pose a challenge. A recent study suggested that integrating dermal matrices with skin grafts may help reduce the severity of scarring and improve overall aesthetics [13]. Such advanced grafting techniques could be explored further in future studies to validate their efficacy in syndactyly repair.

Functionality is the ultimate goal of syndactyly correction, as impaired hand function severely limits daily activities. In this study, 90% of patients regained full dexterity, allowing them to perform tasks appropriate for their age. This finding aligns with other reports that emphasize the importance of early surgical intervention for optimal functional recovery [14]. The remaining 10% of patients experienced mild limitations, likely due to the severity of their syndactyly or other congenital abnormalities. Complex syndactyly cases, involving both soft tissue and bone fusion, often require staged procedures to achieve full functional recovery [15]. Despite the limitations, these patients still experienced significant improvements compared to their preoperative state, reinforcing the importance of surgical separation [16].

Although early intervention is often recommended for syndactyly surgery to enhance functional recovery and minimize complications, ideally between 6 and 18 months [17], our patient cohort did not receive surgery at such an early age. Despite this, our findings indicate that older patients can still achieve significant improvements in web spacing and aesthetics. For complex cases, a slightly delayed approach offers the advantage of thorough preoperative planning, enabling surgeons to customize the procedure to address specific anatomical challenges effectively [18]. Effective postoperative care is paramount in preventing complications such as necrosis and infection. Protective splinting, regular wound monitoring, and structured rehabilitation programs ensure proper healing and minimize the risk of contracture [19]. Our study reinforces the importance of these measures, particularly in reducing the need for secondary procedures. However, more research is needed to standardize postoperative care protocols for syndactyly patients, as current practices vary widely [20].

This study’s retrospective design provided valuable insights, but it has some limitations. The sample size was limited, and the follow-up period varied among patients, potentially introducing biases in assessing long-term outcomes. Additionally, while the study evaluated various flap techniques such as Z-plasty, dorsal base flap, and random flap, it did not directly compare the effectiveness of each method. Future studies should aim for larger, multicenter analyses with longer follow-up periods to comprehensively assess the long-term success of different surgical techniques. The integration of advanced grafting materials and regenerative therapies could also be explored to further improve aesthetic outcomes and minimize scarring. Finally, continued efforts are needed to standardize postoperative care protocols, ensuring all patients receive optimal support for recovery.

## Conclusions

In summary, this study demonstrates that flap and graft techniques effectively improve the functional and aesthetic outcomes of congenital syndactyly surgery. Early intervention, tailored surgical approaches, and comprehensive postoperative care are essential to achieving optimal results. Further research should refine these techniques and protocols to ensure the highest quality of care for affected children. Additionally, the long-term follow-up and monitoring of patients can provide more insights into the effectiveness of surgical techniques and their impact on patients’ quality of life.

## References

[1] Jordan D, Hindocha S, Dhital M, Saleh M, Khan W (2012). The epidemiology, genetics and future management of syndactyly. Open Orthop J.

[2] Braun TL, Trost JG, Pederson WC (2016). Syndactyly release. Semin Plast Surg.

[3] Xing SG, Tang JB (2019). Extending applications of local anesthesia without tourniquet to flap harvest and transfer in the hand. Hand Clin.

[4] Widerberg A, Sommerstein K, Dahlin LB, Rosberg HE (2016). Long-term results of syndactyly correction by the trilobed flap technique focusing on hand function and quality of life. J Hand Surg Eur Vol.

[5] Dao KD, Shin AY, Billings A, Oberg KC, Wood VE (2004). Surgical treatment of congenital syndactyly of the hand. J Am Acad Orthop Surg.

[6] Sood RF, Irwin TJ, Taghinia AH (2022). Syndactyly release in the hand: surgical technique. Plast Reconstr Surg.

[7] Yildirim C, Sentürk S, Keklikçi K, Akmaz I (2011). Correction of syndactyly using a dorsal separated V-Y advancement flap and a volar triangular flap in adults. Ann Plast Surg.

[8] Little KJ, Cornwall R (2016). Congenital anomalies of the hand--principles of management. Orthop Clin North Am.

[9] Vekris MD, Lykissas MG, Soucacos PN, Korompilias AV, Beris AE (2010). Congenital syndactyly: outcome of surgical treatment in 131 webs. Tech Hand Up Extrem Surg.

[10] Mahindroo S, Tabaie S (2023). Syndactyly in the pediatric population: a review of the literature. Cureus.

[11] Mericli AF, Black JS, Morgan RF (2015). Syndactyly web space reconstruction using the tapered M-to-V flap: a single-surgeon, 30-year experience. J Hand Surg Am.

[12] Deunk J, Nicolai JP, Hamburg SM (2003). Long-term results of syndactyly correction: full-thickness versus split-thickness skin grafts. J Hand Surg Br.

[13] Balakrishnan G, Vijayaragavan S, Balakrishnan S (2022). Omega flap technique: revisiting conventional wisdom. Hand (N Y).

[14] Eskandari MM, Oztuna V, Demirkan F (2004). Late psychosocial effects of congenital hand anomaly. Hand Surg.

[15] Mende K, Watson A, Stewart DA (2020). Surgical treatment and outcomes of syndactyly: a systematic review. J Hand Surg Asian Pac Vol.

[16] Lumenta DB, Kitzinger HB, Beck H, Frey M (2010). Long-term outcomes of web creep, scar quality, and function after simple syndactyly surgical treatment. J Hand Surg Am.

[17] Jester A (2023). Timing of surgery in congenital hand differences: a consensus proposal. J Hand Surg Eur Vol.

[18] Wang AA, Hutchinson DT (2019). Syndactyly release: a comparison of skin graft versus graftless techniques in the same patient. J Hand Surg Eur Vol.

[19] Patel NK, Toyoda Y, Grunzweig KA, Shah AS, Mendenhall SD (2023). Recent advancements in the diagnosis and treatment of congenital hand differences. J Am Acad Orthop Surg.

[20] Canizares MF, Feldman L, Miller PE, Waters PM, Bae DS (2017). Complications and cost of syndactyly reconstruction in the United States: analysis of the Pediatric Health Information System. Hand (N Y).

